# Comparison of second harmonic generation from cross-polarized double-resonant metasurfaces on single crystals of Au

**DOI:** 10.1515/nanoph-2021-0677

**Published:** 2022-01-21

**Authors:** Yusuf B. Habibullah, Teruya Ishihara

**Affiliations:** Department of Physics, Tohoku University, Graduate School of Science, 6-3, Aramaki-Aoba, 980-8578, Sendai, Japan

**Keywords:** double resonance, metasurfaces, nonlocal response, second harmonic generation

## Abstract

Second harmonic generation (SHG) from metasurfaces consisting of square array of split ring, heptagon and triangle cross-polarized double resonant resonators, is investigated both experimentally and numerically. The structures are fabricated on single crystalline Au plates using Focused Ion Beam technique. Array of the triangular structure exhibits most efficient SHG. Experimental observation is explained reasonably well by theoretical evaluation of SHG using the overlapping integral of nonlinear polarization and the microscopic field distribution on the metal surface at the SHG frequency taking phase relation between the two fields into account.

## 1 Introduction

Second harmonic generation (SHG) is one of the 2nd order optical nonlinear effects, where two photons with angular frequency ω are combined to generate one with 2ω . Conversion efficiency of SHG is generally so weak that conventionally a large size of crystals under the phase matched condition is used for practical purposes. For nanoscale nonlinear application where it cannot rely on the propagation length, however, an alternative approach is required to make the nonlinearity itself larger.

Metasurface is an ultra-thin artificial structure, which was initially discussed in microwave region [1], [2], [3]. In the spectral range of optics, it was initially considered as a prototype of three-dimensional metamaterials. When it was recognized that the abrupt phase jump plays a significant role for controlling wave propagation; however, it was understood that bulk properties such as permittivity and permeability are no longer necessary [4, 5]. Later it turned out that various novel optical functions including nonlinear properties can be realized by sub-wavelength-thick structure. By appropriately designing the sub-wavelength structures, we have chances to realize the best artificial structure for effective SHG materials. As it is essential to break the inversion symmetry, artificial structures were designed to demonstrate SHG from noncenytrosymmetric materials [6], [7], [8], [9], [10], [11], [12]. In 2006 it was claimed that SHG in a split ring resonator (SRR) was greatly enhanced due to its characteristic magnetic resonance [13]. Later the same group showed that its complementary structure (SRR hole in metallic film) with no magnetic resonance had similar intensity of SHG [14]. In 2015, O’Brien et al. [15] introduced an overlap integral of nonlinear polarization and SHG field to estimate SHG amplitude in the far field. By comparing SHG intensity from SRR metamaterials with different aspect ratios, they show that metamaterials are free from the limit of so-called Miller’s rule [16, 17], which predicts a nonlinear optical susceptibility of a material from its linear ones. The overlap integral based on Lorentz reciprocity was originally discussed by Roke et al. in 2012 for nonlinear scattering processes in colloidal solution [18]. Note that by using the overlap integral, it is naturally understood that SHG vanishes unless overall inversion symmetry is broken, irrespective of type of interactions. SHG from complementary structures was discussed in [19].

In order to enhance SHG efficiency, utilizing shape dependent resonance is also important. By utilizing degree of freedom of structural design, quite a few works have been reported [20], [21], [22], [23]. We have now some excellent review articles in SHG in metallic nanostructures [24], [25], [26].

In this study, we investigate metasurfaces that generate efficient SHG from double resonant isolated Au resonators. We select the fundamental wavelength region of 1100–1500 nm so that the SH field can enjoy plasmonic field enhancement for Au.

To ascertain the best geometry for Au-double resonant metasurface (Au-DRM), we designed and fabricated three geometries, which will be referred to SRR, Heptagon and Triangle, respectively, in this paper. The three Au-DRMs were designed to have resonances at cross polarized excitation, with large nonlinearity achieved through good spatial mode matching of the optical near field on the surfaces of the individual resonators within the metasurfaces at cross polarized resonance. The designs were achievable by taking the full advantage of the two degrees of freedom available at cross polarization, this flexibility removes the need for multi-constituent antenna to achieve double resonance in our design.

## 2 Methods

### 2.1 Metasurface design

The optical properties of the plasmonic nanostructure are modeled by solving Maxwell equations with the periodic boundary condition and the port boundary condition set to constrain the modeled geometry using finite element method (FEM).

**Figure 1:**
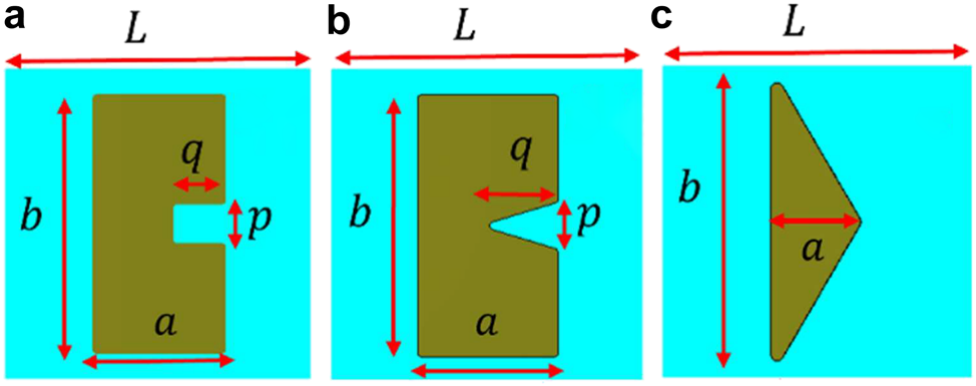
Definition of parameters for (a) SRR Au-DRM. (b) Heptagonal Au-DRM. (c) Triangular Au-DRM.

In order to search for efficient SHG metasurfaces, our first choice is SRR, an orthodox metaatom with a magnetic resonance. As shown in Figure 1a, the shape lacks inversion symmetry in *x*-direction, which guarantee the generation of SHG. In terms of symmetry, nonzero components are $\chi_{xxx}^{\left(2\right)}$ and $\chi_{xyy}^{\left(2\right)}$ . As the effective length for resonance is much longer for *y*-excitation than *x*-excitation, we expect most efficient SHG in $\chi_{xyy}^{\left(2\right)}$ configuration under the double resonant condition. Systematic protocol to find a double resonance condition for orthogonal polarizations was discussed by Habibullah and Ishihara in Metamaterials2020.

As a variant of sprit ring resonator, we chose a Heptagon metaatom (Figure 1b), as the electric field tends to concentrate at the two arms toward right, more efficient SHG was expected at the beginning. As another candidate we chose Triangle metaatom (Figure 1c). While the previous two structures can be obtained by taking a center part away from a rectangular platelet, the third metaatom, Triangle, is obtained by cutting its two corners of one side. For triangular resonator; however, as it is simpler shape than the folded geometry such as SRR and heptagon, it is difficult to find any appropriate parameters to satisfy the double resonance condition. Therefore, we compromised to use the larger unit cell size and the second lowest resonance (with smaller electric field enhancement) as a substitute.

The characteristic structural parameters to achieve a double resonance are summarized in Table 1. The parameters were found after systematic parameter optimization in frequency domain using a commercial software (CST Studio Suite).

**Table 1:** Structural parameters (nm) for double resonant metasurfaces.

	a	b	d	p	q	L
SRR	172	330	40	50	68	400
Heptagon	180	330	40	60	80	400
Triangle	145	490	32			500

### 2.2 Sample preparation

**Figure 2:**
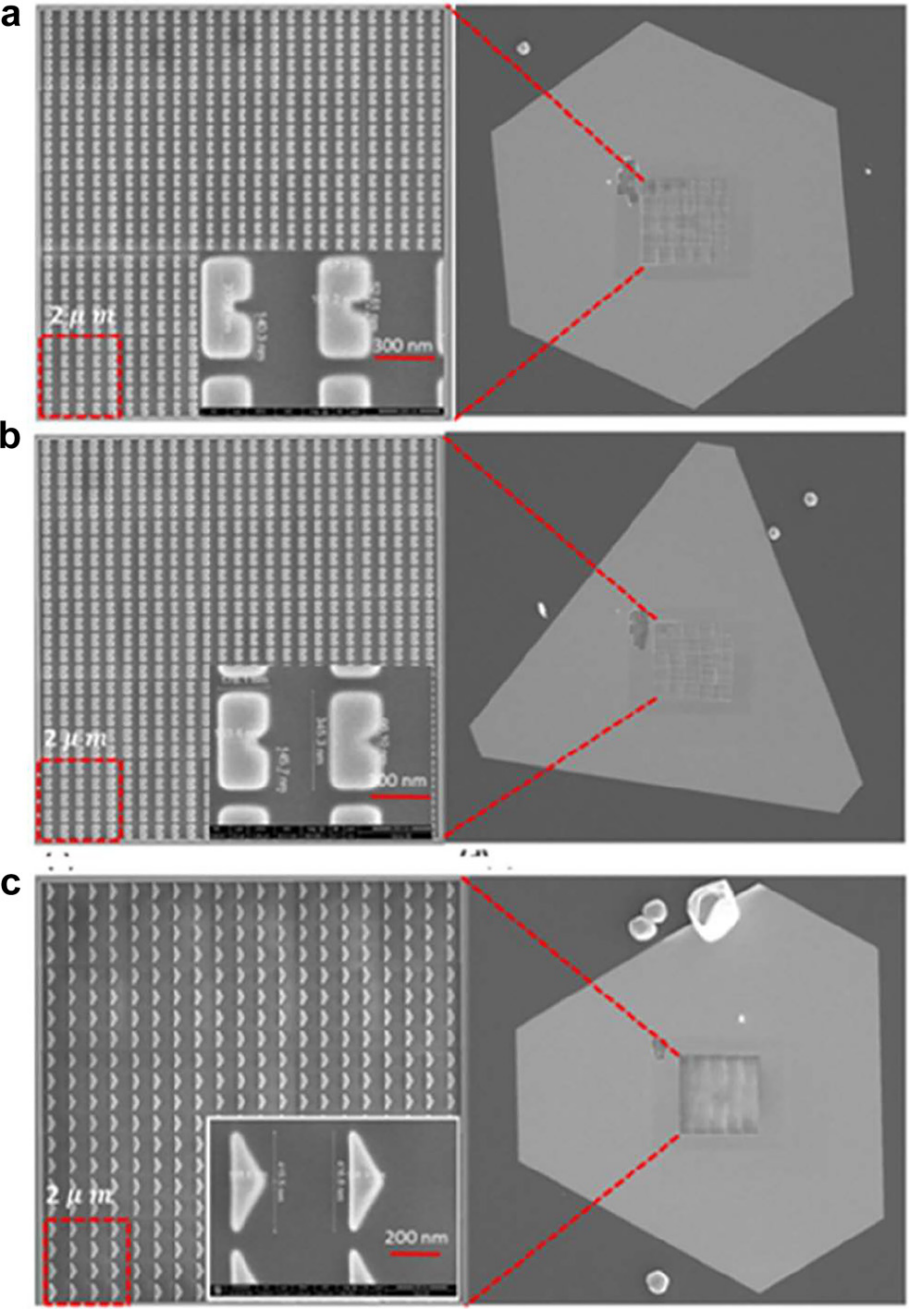
The single crystalline Au flake that is grown chemically on a quartz substrate, the zoomed area is showing the quality of the nanostructure fabrication on it. The nanostructure with area of 10×10 μm2 is showing 25 sections of the four steps fabrication, each having an area of 2×2 μm2 , as shown with the square shape with red broken lines. (a) SRR Au-DRM. (b) Heptagonal Au-DRM. (c) Triangular Au-DRM.

Single crystals of Au were chemically synthesized on quartz substrates basically following the procedure in [27] with some modification described in Supplementary Section S1. Square array of SRR, Heptagon and Triangle resonators were fabricated with Focused Ion beam as are shown in Figure 2. Well-developed facets suggest good quality of single crystals. Metasurfaces with much better quality are prepared on single crystals than sputtered films, which is the key for reproducible measurement. The details of FIB fabrication process is described in the Supplementary Section S2.

### 2.3 Experimental set-up for SHG measurements

**Figure 3:**
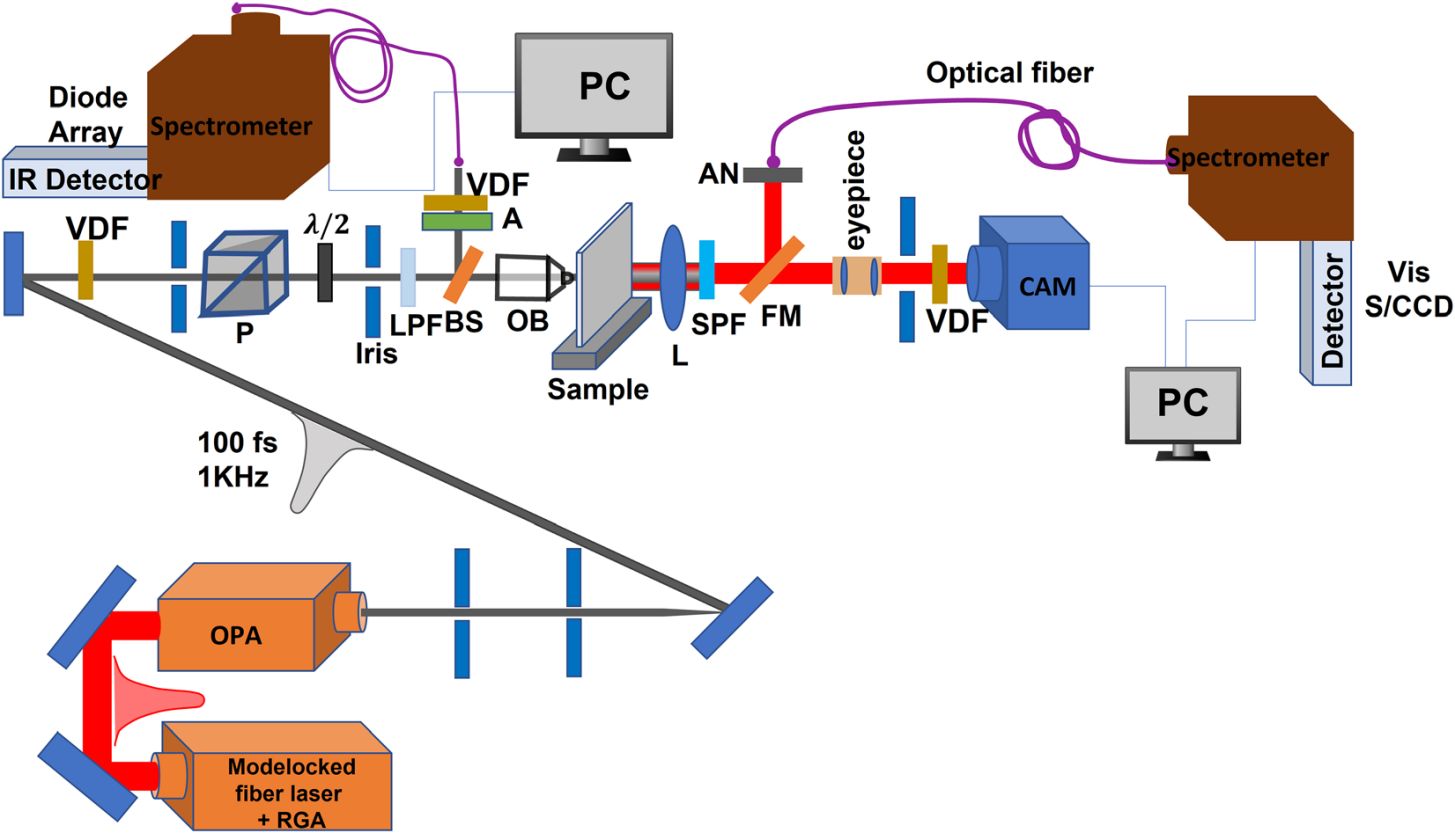
Schematics of the non-linear experimental set up for spectrally resolved second harmonic generation from Au-DRMs (OPA, optical parametric amplifier; S/CCD, spectrometer coupled with charge coupled devices; A, attenuator; VDF, variable density filter; RGA, regenerative amplifier; CAM, infrared camera; OB, objective lens; L, lens; LPF/SPF, long pass filter/short pass filter; FM, flip mirror; PM, power meter; P/AN, polarizer/analyzer).

In this study, SHG generation from Au-DRMs was investigated as a function of excitation wavelength around the double resonance. As a tunable light source, we employed an optical parametric amplifier (OPA) of 100 fs pulse at 1 kHz repetition rate pumped by regenerative amplified Ti:Sapphire laser. The tuning range for the idler is 1000–1600 nm, while the double resonance is achieved for the fundamental wavelength of about 1360 nm. The schematic of the setup is as shown in Figure 3. Details of the setup is described in the Supplementary Section S2.

## 3 Experimental results

**Figure 4:**
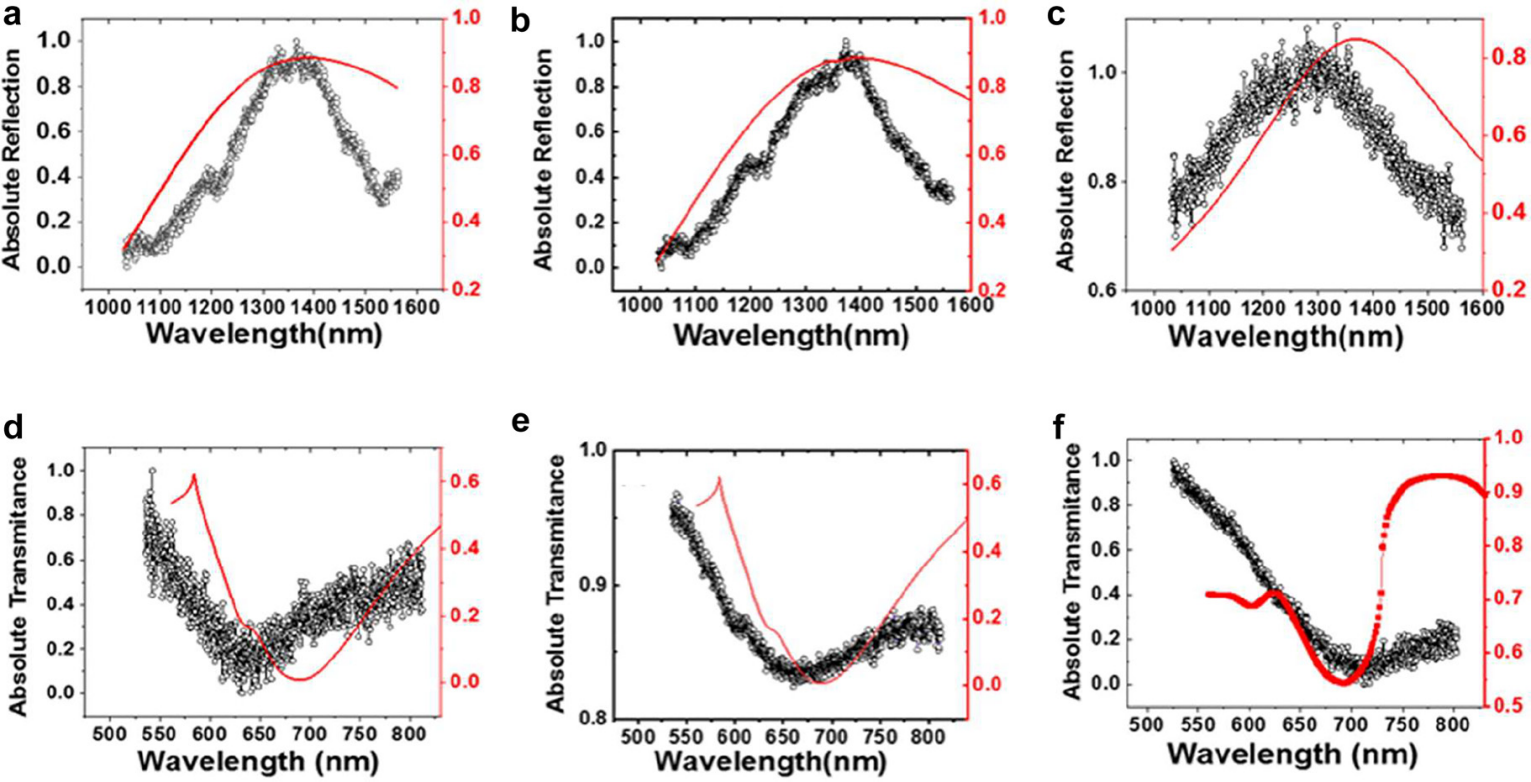
Comparison between simulated and measured absolute reflection spectra of (a) SRR Au-DRM (b) heptagonal Au-DRM (c) SRR Au-DRM for *y*-polarization in the fundamental wavelength region. Comparison between simulated and measured absolute transmission spectra of (d) SRR Au-DRM (e) heptagonal Au-DRM (f) SRR triangular Au-DRM for *x*-polarization in the SHG wavelength region.

**Figure 5:**
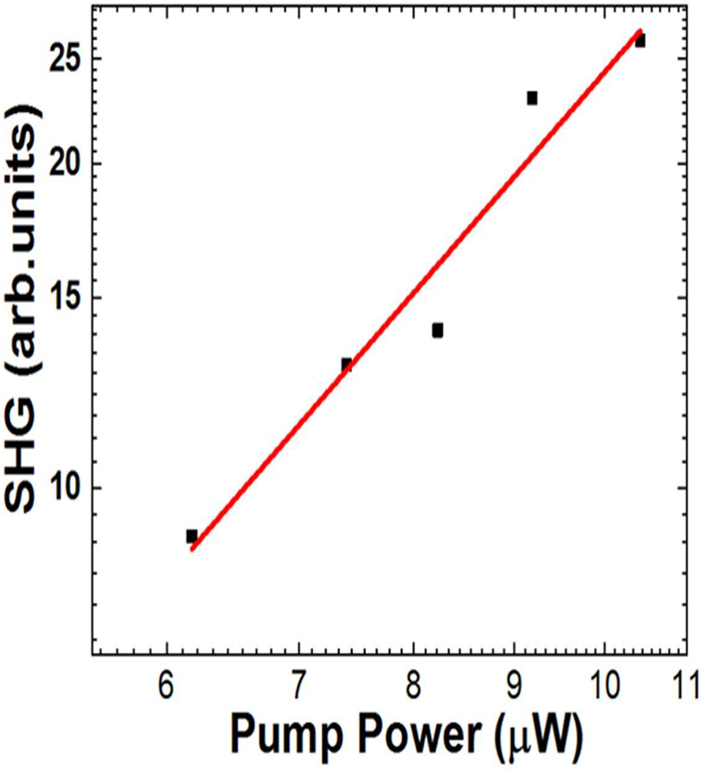
Excitation power dependence. The fundamental excitation is *y*-polarization at resonant wavelength of 1360 nm and the SHG emission was detected for *x*-polarization at 680 nm.

**Figure 6:**
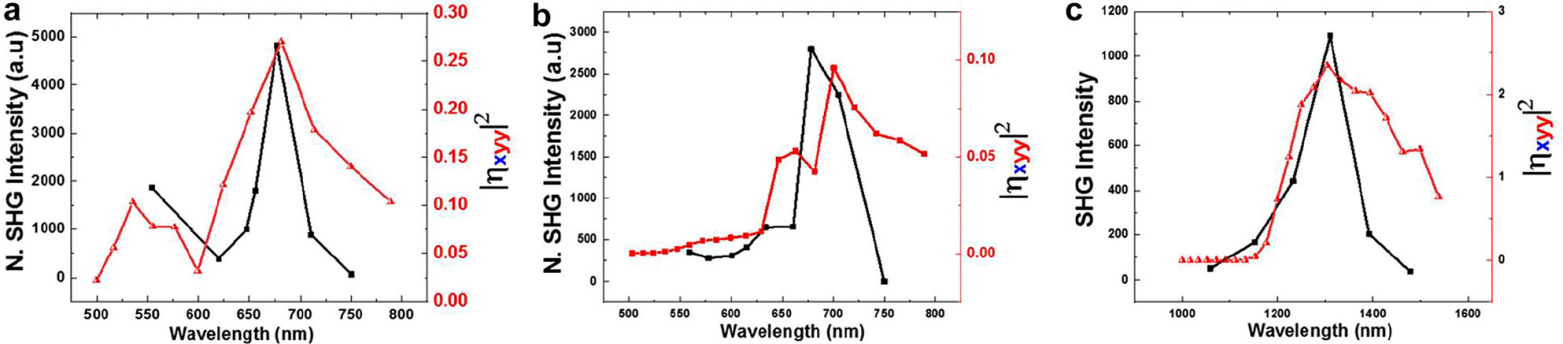
Comparison of SH intensity wavelength dependence between experiment and finite element simulation for (a) SRR Au-DRM. (b) Heptagonal Au-DRM. (c) Triangular Au-DRM. The black dots are experimental data points, while the solid curves are the guide to the eye. The red solid curve is fit for the FEM simulation data (red square dots). The incident excitation is *y*-polarization and the SHG emission was detected for *x*-polarization.

Prior to the SH excitation spectroscopy, we measured the far field linear responses to characterize our metasurfaces. The reflection spectra for *y*-polarized excitation (normalized by flat Au film) are shown in Figure 4a–c with simulation results (red curves). Based on simulation, the reflection peak is ascribed to the lowest mode (without any nodes in electric field distribution) of these samples. Note that not only the resonance wavelength but also their widths are fairly well reproduced by our numerical simulation with periodic boundary condition. It demonstrates that the width is not due to the inhomogeneous broadening from the sample fabrication fluctuation, but intrinsic to the system. The relatively large width suggests the large light–matter interaction of the transition. Figure 4d–f shows transmission spectra (normalized by flat Au film) with simulation results (red curves). Again, the wavelength of transmission minimum and its band width are reproduced by the simulation. Therefore, we safely conclude that our metasurfaces are prepared as designed. The SHG emission in the transmission direction was detected for various excitation wavelength around the resonance. The SHG emission after filtering out the fundamental frequency by the short pass filter was detected with CCD spectrometer. As we measure the spectra for each excitation wavelength, we can easily identify the SHG from the sample. Figure 5 shows that the SHG emission is proportional to the square of the fundamental intensity, which is expected for SHG. As the light intensity from OPA varies for different wavelengths, we normalize the SHG intensity based on the square dependence. We plotted the normalized SH intensity as a function of SH emission wavelength depicted in Figure 6, which shows that the SH emission intensity has a strong dependence on the fundamental excitation wavelength. The correlation between the linear responses in Figure 4 and the nonlinear SH response in Figure 6 confirms the presence of strong local field enhancement due to the double plasmonic resonance at the crossed polarization excitation. Note that as experimental conditions are not exactly the same for Figure 6a–c, the counts cannot be compared directly. When we compare the SH intensity at the resonant peak to the off-resonant positions, it is observed that the SH conversion efficiency is several orders of magnitude more at the respective resonant peaks. Such a large local field enhancement is a characteristic of localized surface plasmons in plasmonic nanostructures such as Au-DRMs. This behavior confirms the importance of linear resonances toward achieving large nonlinearity [28], [29], [30]. The red curves are calculated responses, which will be explained later. Recently anisotropic SHG was reported from single crystals of gold [31]. But the differences in orientations of arrays for three structures (observed in Figure 2) are not important in our experiment, as SHG from metasurfaces are overwhelming these SHG from crystal anisotropy.

**Figure 7:**
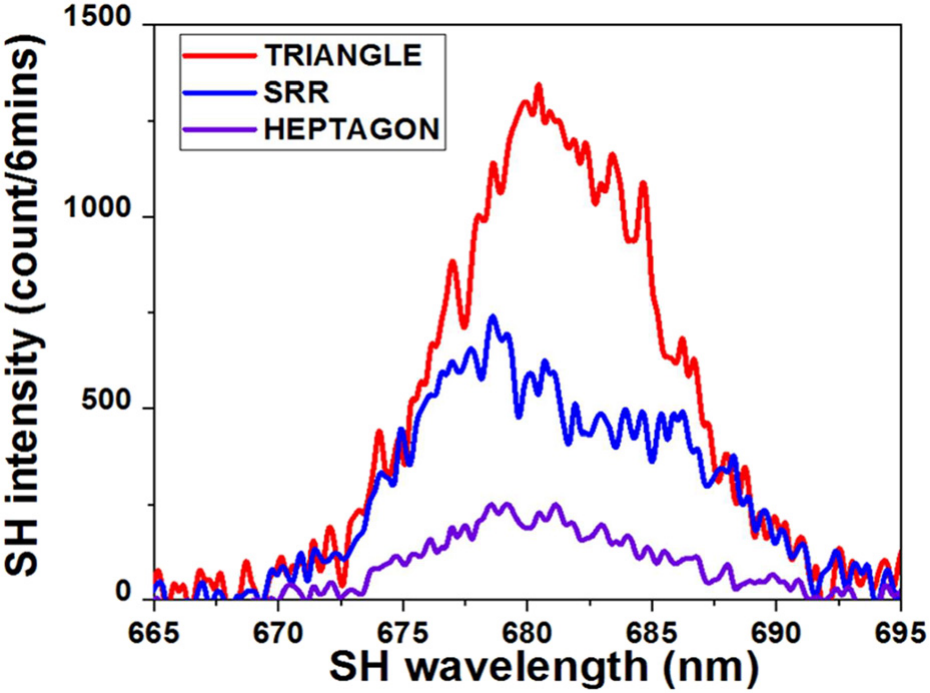
Comparison of transmitted second harmonic spectra for SRR, heptagonal and triangular Au-DRMs under the same experimental condition. The incident polarization is *y*-polarization at fundamental resonant wavelength of 1360 nm and SHG emission was detected for *x*-polarization.

In order to compare SHG efficiency for the three geometries experimentally, we measured the *x*-polarized SH intensity for *y*-polarized excitation at 1360 nm, which corresponds to the resonance for the SRR and Heptagon, but slightly off resonance for Triangle. Three samples were measured successively without changing the experimental conditions, which is shown in Figure 7. The SH intensity of Triangle is 1 order greater than Heptagon and two times greater than SRR Au-DRM in spite of a slightly off resonant condition for Triangle. Combining with wavelength dependence of SHG intensity, we estimate that the Triangle is one order of magnitude more efficient. To evaluate the nonlinear-coefficient of Triangle, which is our most efficient nonlinear metasurface, we use the SHG average power, $P_{\text{SHG}}$ from the count of CCD by using HeNe laser for calibration, the detail is explained under the Supplementary Information S4.

## 4 Theoretical consideration

Once the design of metasurface is defined, it is possible to calculate SHG at the farfield, using the nonlinear polarization distribution at the metal surfaces as a source. An alternative way is to evaluate the overlapping integral as was first applied for matamaterials by O’Brien et al. [15] to demonstrate that the Miller’s rule does not always work to predict a good structure for nonlinear metasurfaces. Since then there are several works, but it seems to our knowledge that all of them fixed the laser wavelength and change the structure. Our approach is to vary the laser wavelength and measure the SHG excitation spectra for different structures.

In our approach, we do not consider quantum-mechanical effects due to the electron density spill-out from the nanoparticles and inter-particle electron tunneling [32, 33]. This is justified, because the separation distances between our nanoparticle is not in sub-nanometer scale but rather in tens of nanometers which guarantees that the electronic densities of our individual nanoparticle do not overlap [34, 35].

To investigate the most efficient Au-DRM for SHG generation, we evaluated the nonlinear coefficient of the metasurfaces from the overlapping integral [36], with which far field emission is predicted from a nonlinear polarization source. In order to explore it predictive capabilities for arbitrary geometry of any metasurface, we introduced a normalization factor into the overlapping integral to arrive at the non-linear coefficient. Here, the local nonlinear susceptibility tensor is assumed on the surface of the Au metasurface for the description of non-linear emission. More specifically for this study, the second harmonic emission is estimated by assuming a second order nonlinear susceptibility on the surface of metasurface. The second order nonlinear emission is evaluated from a theory based on Lorentz reciprocity as

(1) $$E^{SHG}\propto \iint E_{⊥}^{2\omega }\left(r\right)P_{⊥}^{\left(2\right)}\left(r\right)\text{d}S$$

where $E_{⊥}^{\text{SHG}}$ is an electric field amplitude of the second order non-linear emission in the far field, $E_{⊥}^{2\omega }\left(r\right)$ is the normal component of a hypothetical local electric field at 2ω frequency due to second harmonic wave propagating from the detector position back to the metasurface, d*S* is spanned on the surface characteristics of the second-order nonlinear susceptibility and ${\text{P}}_{⊥}^{\left(2\right)}\left(r\right)$ is the normal component of the second order nonlinear polarization at position r on the surface.

For a given fundamental field, electric fields at metasurfaces can be calculated numerically. In order to achieve fair comparison of nonlinearity between arbitrary geometry of any metasurface, we formulated the nonlinear coefficient by introducing a normalization factor, which involves normalizing Eq. (1) by the cube of the amplitude of incident electric field and area of the unit cell of the periodic structure. The nonlinear coefficient is given by

(2) $$\eta =\frac{\iint E_{⊥}^{2\omega }\left(r\right)E_{⊥}^{\omega }\left(r\right)E_{⊥}^{\omega }\left(r\right)}{E_{0}^{3}}\frac{\text{d}S}{L_{x}L_{y}}$$

where $E_{0}$ is the amplitude of the incident electric field and $L_{x}L_{y}$ is the unit cell area.

Comparing our experimental results to theoretical estimation of SHG intensity from the overlapping integral, we have discovered an inherent ambiguity of the overlapping integral method. As the excitation at the fundamental frequency and the plane wave from the detector back to the sample are calculated independently, the phase relation between the two waves can be set arbitrary. The overlapping integral, however, depends on the relative phase as is shown in Figure 9. This is an inherent problem of the overlapping approach: In reality, it is the excitation wave at fundamental frequency that generates second order nonlinear polarization at the surface. The phase relation between the polarization and SHG wave is naturally determined in the inhomogeneous wave equation with the nonlinear polarization as a source term. On the other hand, in the overlapping integral approach, the two waves are calculated independently. The phase relation between the two waves cannot be found in advance of calculation of the overlapping integral. We adopt the right phase delay that gives the largest overlapping integral. Note that the phase delay is frequency dependent. As far as we know, this ambiguity has never been discussed in previous literature.

Now let us consider how we implement the numerical calculation to the formula. Electric field induced by fundamental and hypothetical light from the observation oscillates in time with angular frequency ω and Ω=2ω , respectively.

Therefore the integrand is

$$\left(E_{\Omega }^{\prime }\left(r\right)\text{cos}\left(\Omega t-\delta \right)+E_{\Omega }^{\prime \prime }\left(r\right)\text{sin}\left(\Omega t-\delta \right)\right)\times {\left(E_{\omega }^{\prime }\left(r\right)\text{cos }\omega t+E_{\omega }^{\prime \prime }\left(r\right)\text{sin }\omega t\right)}^{2}$$

$$=\text{Re}\left\{\left|E_{\Omega }\left(r\right)\right|{\text{e}}^{i\left(\Omega t-\beta -\delta \right)}\right\}{\left(\text{Re}\left\{\left|E_{\omega }\left(r\right)\right|{\text{e}}^{i\left(\omega t-\alpha \right)}\right\}\right)}^{2}$$

$$=\frac{\left|E_{\Omega }\left(r\right)\right|{\left|E_{\omega }\left(r\right)\right|}^{2}}{8}\left({\text{e}}^{i\left(2\alpha -\beta -\delta \right)}+{\text{e}}^{-i\left(2\alpha -\beta -\delta \right)}+\text{oscillating terms}\right)$$

$$\to \frac{\left|E_{\Omega }\left(r\right)\right|{\left|E_{\omega }\left(r\right)\right|}^{2}}{4}\text{cos}\left(2\alpha \left(r\right)-\beta \left(r\right)-\delta \right),$$

where α(r) and β(r) are phase at r for the fundamental and hypothetical field, respectively, and δ is a relative phase delay for the two waves. The oscillating terms due to the optical rectification or photo galvanic effects, which are out of our scope. If we choose δ to be δ=α−β , the value would be the largest, but actually α and β are dependent on r. Therefore for fixed δ, cos(2α(r)−β(r)−δ) can be positive or negative depending on the position. As the SHG emission should be coherent to effective polarization of the structure, we should choose δ to make the integral maximum.

The nonlinear coefficients were numerically calculated using

(3) $$\eta_{\mathrm{xyy}}=\frac{1}{E_{0}^{3}L_{x}L_{y}}\iint \text{abs}\left(E_{⊥}^{x,2\omega }\left(r\right)\right)\text{abs}\left(E_{⊥}^{y,\omega }\left(r\right)\right)\text{abs}\left(E_{⊥}^{y,\omega }\left(r\right)\right)\text{cos}\left(2\alpha \left(r\right)-\beta \left(r\right)-\delta \right)\text{d}S$$

where electric fields are evaluated 3 nm away from the upper surface (side boundaries) of the Au-DRMs d*S* is the area over which the normal component of the local electric field was calculated. Further details about the numerical simulation is explained in Supplementary Information S5.

## 5 Discussion

**Figure 8:**
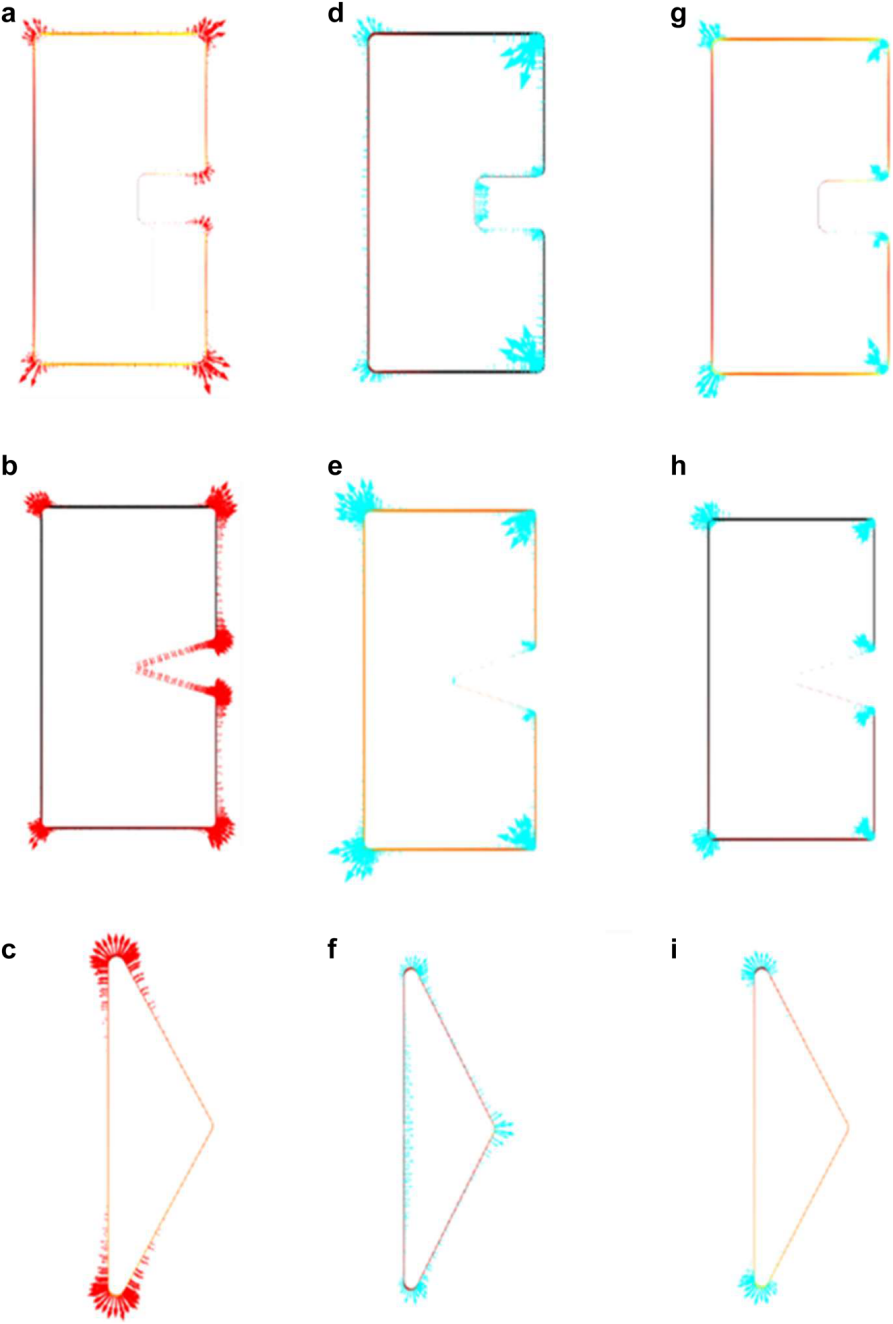
Surface arrow plot showing the symmetry of squared normal component of local electric field enhancement distribution at a resonant position 1360 nm upon *y*-polarization excitation of (a) SRR (b) Heptagon (c) Triangle. Corresponding plot for *x*-polarization at a resonant position 680 nm (d)–(f). Corresponding plot for product of the two (g)–(i), representing the local SHG.

**Figure 9:**
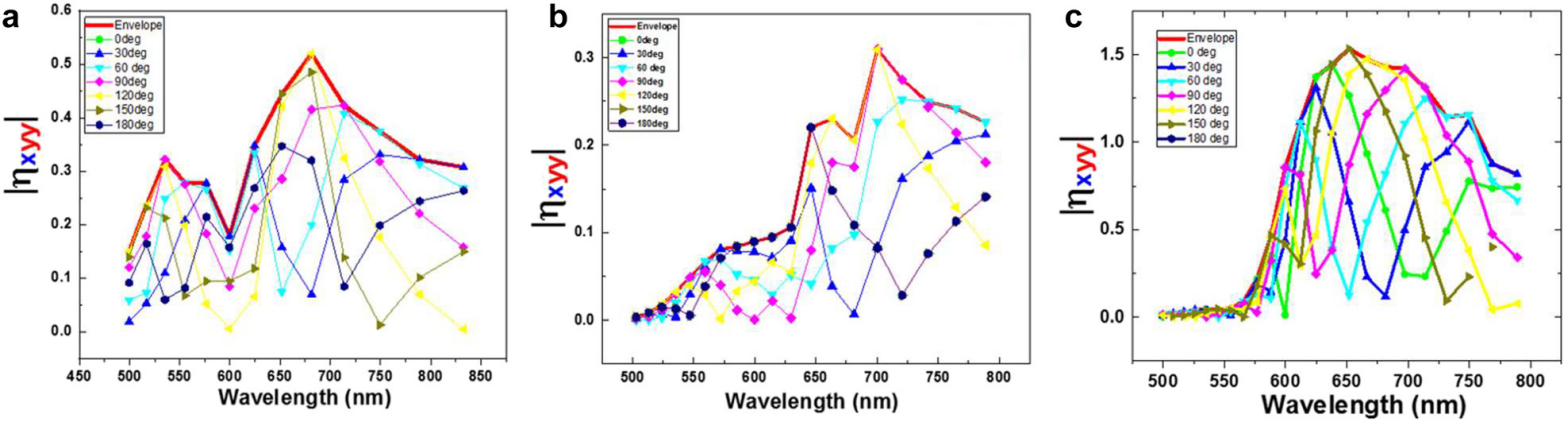
(a) Relative phase dependence of nonlinear coefficient as a function of wavelength for (a) SRR Au-DRM. (b) Heptagonal Au-DRM. (c) Triangular Au-DRM. The thick line in each graph corresponds to the upper envelope of the phase dependence of nonlinear coefficient as a function of wavelength.

In order to identify the best geometry for Au-DRM that foster efficient spatial mode matching at cross polarized double resonant wavelength, the vectorial electric field distribution at fundamental and SH emission wavelength was numerically calculated using FEM (COMSOL Multiphysics 5.5 with wave optics module) in a 3D simulation space (see Figure S2 in the supplementary section). In a form of arrow plot the magnitude of the square of the normal component of the electric field $[E_{⊥}^{y,\omega }\left(r\right)$ ]^2^ upon *y*-polarization excitation were evaluated at the fundamental resonant wavelength of 1360 nm as in Figure 8a–c. In a similar fashion, the magnitude of the normal component of the electric field $E_{⊥}^{x,\omega }\left(r\right)$ upon *x*-polarization excitation were evaluated at the SH emission wavelength of 680 nm as is in Figure 8d–f. The evaluation was carried-out only on the surface that contributes to SHG from a symmetry point of view. The integrand which is the product of the two fields is shown in Figure 8g–i. Note that in Figure 8g and h the integrand has different signs which cancel to each other in the integral. On the other hand, in Figure 8i the integral is always toward outside, which means that they contribute to the integral without canceling. That is the reason of superior efficiency for SHG generation of triangle metasurface. Interestingly as is shown in Figure 8h, the excitation with *x*-polarized light, which mainly occurs in the middle of the triangle, produces polarization to *y*-directions at the upper and lower end of two acute angles. This nonlocal response will be discussed in a separate paper [37]. Figure 9 shows overlapping integral for various relative phase delay of fundamental and hypothetical fields for the three metasurfaces. Note that for a particular phase delay, the efficiency sensitively changes depending on the wavelength. The thick red curves are envelope of the set of the curves, which is the actual SHG efficiency to be compared with the experiment, as were shown in Figure 6. The envelope curves reproduce the experimental findings reasonably well.

## 6 Conclusions

We have investigated wavelength dependence of SHG from doubly resonant metasurfaces consisting of array of SRR, Heptagon and Triangle fabricated in single crystals of Au. One order of magnitude of superiority in SHG efficiency is found in the Triangle metasurface. Numerical calculation based on the overlapping integral augmented with phase delay optimization explains successfully the experimental observation. Our results contribute to the understanding of the linear and nonlinear optical properties of double resonant metasurfaces and open the way for the efficient nanoscale nonlinear medium with potential application in photonic integrated nanocircuitry and nanooptoelectronics.

## Supplementary Material

Supplementary Material Details
