# Supplementary material for: Comparison of second harmonic generation from cross-polarized double-resonant metasurfaces on single crystals of Au
Source: Nanophotonics. 2022 Jan 21;11(9):1931–9. doi: 10.1515/nanoph-2021-0677 (PMC11501336; doi:10.1515/nanoph-2021-0677)
Supplement: Supplementary file 1 — Supplementary Material Details [file j_nanoph-2021-0677_suppl.docx]

**Supplementary information**

**Comparison of Second Harmonic Generation**

**from cross- polarized double-resonant metasurfaces**

**on single crystals of Au**

Yusuf B. Habibullah and Teruya Ishihara

***Corresponding author: Teruya Ishihara**, Tohoku University, E-mail: [t-ishihara@tohoku.ac.jp](mailto:t-ishihara@tohoku.ac.jp)

**Yusuf B. Habibullah:** Tohoku University, E-mail: [khaalidah22@gmail.com](mailto:khaalidah22@gmail.com)

**S1. Preparation procedure for single crystalline Au flake growth**

The growth of single crystalline Au flakes was carried out following the procedure in Ref. [1], with some modification to the time duration of the growth. The growth mechanism is also discussed in details in the aforementioned reference. The procedures can be summarized in 5 steps, as sequentially depicted in Fig. (S1). The first step involves the cleaning through ultrasonic bath of the quartz substrate with acetone and ethanol for 10 minutes in each. Afterwards, the substrate is rinsed in ultrapure water, and then the left over water droplet is blown by nitrogen gas, after which it is dried on a hot plate at 120 ℃. The substrate is then exposed to oxygen plasma for 15 min. The acceleration voltage was 1000 V at microwave power of 100 W and the set oxygen flow rate was set at 1 sccm. The second step requires the preparation of a solution needed for the growth of the flakes, which involves preparing 0.5 M aqueous solution of Hydrogen tetrachloroaurate(III) Trihydrate (HAuCl_4_, 058-00986, FUJIFILM Wako Pure Chemical Corporation). Subsequently, 50 $\mu L$ of the prepared aqueous solution of HAuCl_4_  was then dissolved in 25 mL ethylene glycol (EG, 073-00933, FUJIFILM Wako Pure Chemical Corporation) as a solvent, the resulting mixture was then uniformly mixed under ultrasonic bath for five minutes resulting into a growth solution with 1mmol/L of HAuCl_4_. In the third stage, the cleaned substrate was then attached to a glass holder with a double sided Kapton tape, such that the tape covers one face of the substrate entirely. This ensures that the growth of the flakes is limited to only one face and not to the other face of the substrate. The glass holder with the attached substrate is then inserted into a polypropylene centrifuge tubes (Violamo, 50 mL), after which the resulting solution from step 2 is poured into the tube to a level that submerges all the substrates within the tube. In the fourth step, the tube was closed and transferred into an oven set at 90 ℃ to kick start the growth reaction. The tube was positioned vertically to avoid small particle contamination and aggregation.


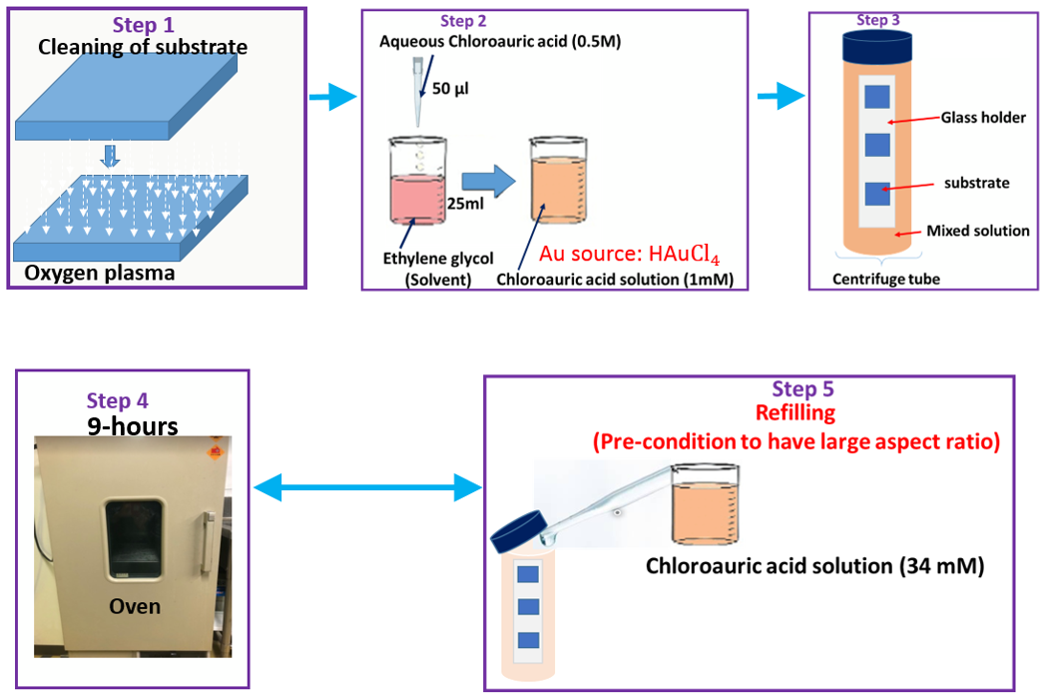


Fig. S1 Schematic illustration of substrate cleaning, solution preparation, growth positioning, controlled heating and refilling process.

**
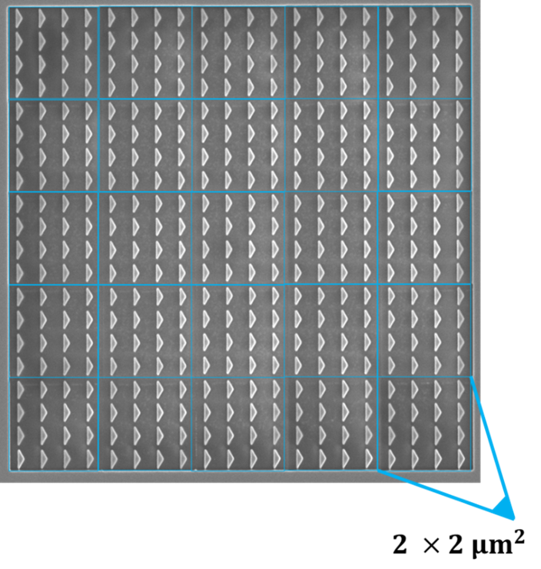
**

Fig. S2 Triangular Au-DRM showing 25 sections of the four steps fabrication, each having an area of ${2 \times2 \mu m}^{2}$.

**S2. Sample fabrication**

The surface chemical stability and non-toxicity of gold makes it a plasmonic material of our choice. Achieving double resonant plasmonic nanostructure requires high degree of accuracy in fabrication, and this is why most of the research on double resonant nanostructures have been limited to single antenna rather than array of antenna. Thanks to FIB fabrication method where uncertainty below 10 nm can be achieved under lowest ion beam aperture[2]. In addition, perfection in fabrication is crucial for achieving the double resonance nanostructure, which necessitates the use of single crystalline Au metal that guarantees extremely crucial reproducible geometries, especially for large array of nanostructure.

Plasmonic nanostructure are usually fabricated using electron beam lithography(EBL)[3] or focused ion beam milling (FIB)[4]. In our case we employ FIB for the nanostructure fabrication because of the fabrication accuracy required to achieve double resonant metasurfaces as well as its site-specific capabilities. We employ dual beam FIB/SEM microscope FEI Helios (Versa3D) using gallium ions with energy of 30 kV and ion beam current of 10 pA. The energy and the current were optimized to achieve the best spatial resolution of the milling. The nanostructure was fabricated on a single crystalline gold flake grown on a quartz substrate (See Fig. 2 in the main text), following the procedure in [1]. From the well-developed facets, it is clear that they are good single crystals. This procedure makes EBL fabrication method incompatible to be used, since it requires strict chemical procedure such as resist coating, development and lift up [5]. Prior to our fabrication using FIB, sample was coated with carbon coating layer of 5 nm, this is necessary to avoid charge accumulation during FIB milling. In order to make the fabrication time reasonable using FIB, we limited the nanostructure field to maximum of ${10 \times10 \mu m}^{2}$ with an average fabrication time of 1 hour. To improve the quality of the fabrication, we divided the fabrication of ${10 \times10 \mu m}^{2}$ into 25 steps. We fabricated ${2 \times2 \mu m}^{2}$ (see Fig. 2 in the main text, with the square shape with red broken lines) twenty-five times to make an array of $5 \times5$ as is in Fig. S2, by so doing we ended up with ${10 \times10 \mu m}^{2}$ (see the zoomed part of Fig. 2, in the main text) size nanostructure. The four-step fabrication was done while maintain the periodicity in the best possible way. This stepwise procedure is necessary because focusing of the ion beam milling deteriorates over time. Fabrication of our ${2 \times2 \mu m}^{2}$ nanostructure takes an average of 1 minutes.

**S3. Experimental setup for SHG measurement**

The excitation pulse intensity from the OPA is controlled using Variable Density Filter (VDF). The polarizer (P) and half wave plate ($\lambda/2$) are used to select and rotate (i.e. at constant intensity) the polarization of the linearly polarized incident pulse respectively. The long pass filter (LPF) is used to block the generated SHG from optics before the sample. The beam splitter (BS) split the pulse into the ratio 1:3 (reflected and transmitted). The reflected pulse is for acquiring the center wavelength of the excitation pulse and channeled to the IR spectrometer (Princeton instrument, SP2300i, 1-dimensional array) with InGaAs diode array. The VDF is placed before IR optical fiber to control the intensity of the reflected excitation pulse channeled into the IR spectrometer. The PC connected to the IR spectrometer is used to monitor center wavelength of the excitation pulse during wavelength tuning of the OPA. The transmitted pulse is channeled to the back of the long working distance objective lens (OB) $5\times$ with 0.14 NA. The OB then focus the beam on the sample sitting on a rotational stage, with a spot diameter of 10$\mu m$. The transmitted residual pulse through the sample is focused by the lens (L) on the eyepiece, the real image of the sample is collected from the OB and projected as well as magnified on the IR camera, and eventually yielding the image of the sample on the camera. The use of IR camera to view the sample was necessary due to the micrometer-size dimension of the sample (${10 \times10 \mu m}^{2}$). The average power of excitation of the sample is about 25 $\mu W$.

**S4. Estimation of efficiency**

In order to know the conversion rate of power to count by our visible spectrometer, we employ CW HeNe laser, which has a close wavelength (633 nm) to our emission SHG wavelength (650 nm). Given that the saturation intensity of power meter and CCD is far different, we use four neutral density filters ( 1%, 1%, 1%, and 5 %) to reduce the intensity below the saturation threshold. Separate measurement shows that this set of filters reduces laser power by a factor of 1.1 $\times$ 10^-7^. To determine the equivalent power of emitted SHG just after the sample before passing through any optical element, the power of the CW HeNe laser was measured at the sample position with value of 320$\mu W$ $\times$ (1.1 $\times$ 10^-7^). The equivalent measured count by the CCD was (2 $\times$ 10^6^ c/s), integrated over the line width. From our nonlinear SHG intensity from Triangular Au-DRM measurement we have the count by CCD to a value of 8 $\times$ 10^4^ c/s, integrated over the line width. Therefore, our problem is summarized in the table S1 below

The parameter $x$ can be evaluated from count as

$x=\frac{3.4\times{10}^{-5}}{2.2\times{10}^{6}}7.5\times{10}^{4}$ = $1\times{10}^{-6}$ $\mu W$

Table S2 show the comparison of our achieved nonlinear coefficient from triangular Au-DRM with the previous achieved nonlinear coefficient for plasmonic metasurface at optical frequencies. The conversion efficiency characterizes the experiment, that is the number of fundamental photons needed to generate a single SHG photon. Our evaluated conversion efficiency is 2 orders of magnitude higher than the previous studies of metasurfaces composed of isolated SRR particles as referenced in Table S2. On the other hand, the nonlinear coefficient characterizes the efficiency of metasurfaces in term of their SHG response, of which our triangular Au-DRM has 2 orders of magnitudes higher than the previous studies as shown in Table S2. Their nonlinear coefficients were evaluated from the corresponding parameters given in the referenced papers, while the conversion efficiency was directly provided.

Table S1. Parameter to calculate the power of the SHG intensity at the point of emission

|  | SHG@650nm | HeNe@633nm |
| --- | --- | --- |
| Power@sample [$\mu$W] | $x$ | $3\times{10}^{-5}$ |
| CCD [count /s] | $8\times{10}^{4}$ | $2\times{10}^{6}$ |

Table S2. Comparison of nonlinear coefficient with previous studies

| Item | This work | Klein2007[6] | Feth2008 [7] | Niesler2009 [8] |
| --- | --- | --- | --- | --- |
| Maximum conversion efficiency | $6\times{10}^{-8}$ | $2\times{10}^{-11}$ | $3\times{10}^{-11}$ | $5\times{10}^{-10}$ |
| Peak nonlinear coefficient [$W^{-1}$] | $2\times{10}^{-13}$ | $6\times{10}^{-15}$ | $5\times{10}^{-15}$ | $2\times{10}^{-13}$ |

**S5. Numerical approach to evaluate and compare SHG intensity of arbitrary metasurfaces**

To compare SHG intensity of arbitrary metasurfaces, we disentangle the problem into three steps. The first step is the evaluation of nonlinear polarization from the linear response. Secondly the second harmonic emission is evaluated [6][7][8]. The lastl is the estimation of the nonlinear coefficient. All the FEM simulation presented in this paper were computed in frequency domain, since the time domain versions of FEM are known to be less efficient[9]. The 3D simulation space was composed of embedding space (air), Au nanoparticle and quartz substrate as shown in Figure S2. The objective of the model is to calculate nonlinear coefficient near the double resonance frequency and at off resonance frequency and compare our prediction with the experimental results for three different geometries of the metasurfaces. The two boundary conditions are implemented under full wave formulation option. The periodic boundary condition (i.e. Floquet periodicity) is defined at the the *xz* - and *yz*- planes, while the port boundary condition is defined at the *xy*- planes as shown in Fig. S3. The *y*–linearly polarized plane wave electromagnetic source is from the port 1 at normal incidence through a semi-infinite free space. The *x*–linearly polarized plane wave electromagnetic source is from the port 2 at normal incidence through the quartz substrate defined by refractive index set at 1.46 with the thickness of 500 nm. This is to mimic electric field radiated by a metasurface by sending it back from the detector. COMSOL software allows assigning basic optical properties, such refractive index to a specific simulation space. The dimension of the embedding medium and thickness of the substrate was chosen such that increasing the size further will not affect the simulation results. The *y*–polarized plane wave is excited at $\omega$ frequency , and the generated local electric field distribution is used to evaluate the second order nonlinear polarization $\mathbf{P}_{\perp}^{\left( \mathbf{2} \right)}=\boldsymbol{\varepsilon}_{\mathbf{0}}\boldsymbol{\chi}_{\boldsymbol{S},\perp\perp\perp}^{\left( \mathbf{2} \right)}E_{\perp}^{\boldsymbol{\omega}}\left( \boldsymbol{r} \right)E_{\perp}^{\boldsymbol{\omega}}\left( \boldsymbol{r} \right)$ at each position $\boldsymbol{r}$**,** 3 nm away from the surface of the metasurfaces. Among all the three possible contributing components, only the normal component is evaluated for the computation of the $\mathbf{P}_{\perp}^{\left( \mathbf{2} \right)}$, this is because of its dominance over other components [10]. The *x*–polarized plane wave is excited at $2\omega$ frequency, and the generated local electric field distribution is used to evaluate the normal component of the second harmonic mode $\mathbf{E}_{\perp}^{\boldsymbol{2\omega}}\left( \boldsymbol{r} \right)$oscillating at $2\omega$ at each position $\boldsymbol{r}$along the surface of the metasurfaces. The full wave formulation guarantees the propagation of light at $\omega$ from port 1 to port 2 and vice versa for the propagation of light at 2$\omega$.

In FEM, the entire model (i.e. the nanostructure, the substrate, and superstrate) has to be discretized. The discretization of the model was conducted using the user-controlled meshing option in COMSOL, which partitioned the domains (i.e. volume) and boundaries (i.e. surface) within the simulation space into a collection of free tetrahedral and free triangular finite elements respectively. Due to the large field enhancement resulting from plasmonic double resonances, the meshing must be constrained to ensure convergence of the simulation in the visible and near infrared ($\lambda=400-1600 nm$). The boundaries of the nanoparticle and its interface with quartz substrate were restricted to a maximum element size of 8 nm with maximum element growth of 1.4 and curvature factor of 0.6. The maximum element size is set not to exceed $\delta/2$, where $\delta$ is the skin depth of Au, with value ranging between 28 – 44 nm. This ensures that the fields variation is captured within the skin depth. The other surfaces were set to a maximum element size of 50 nm with similar settings for maximum element growth and curvature factor. The maximum element size is restricted so that it does not exceed $\lambda$ /6 in the dielectric media to ensure that the electromagnetic wave is resolved. The resulting maximum number of parameters required to describe the full field simulation is $2\times{10}^{5}$- $4\times{10}^{5}$ degree of freedom. The computational cost required to compute the nonlinear coefficient for a given periodic geometry required up to 5 -12 hours for each optimization using MUMPS direct solver. The desktop is equipped with Intel (R) Core (TM) i7 CPU operating at 2.67 GHz with 24 GB of RAM and 64-bit single processor.


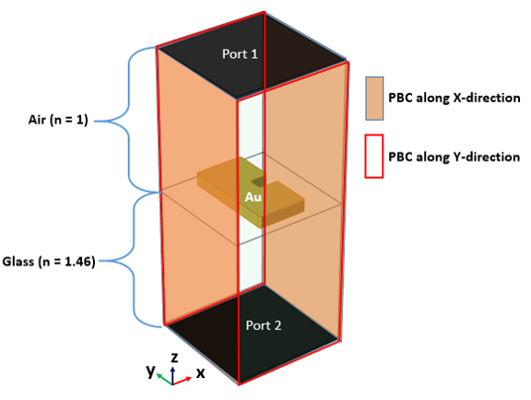


Fig. S3 Schematic illustration of model geometry as implemented for full wave formulation in 3D. PBC – periodic boundary condition.

References

[1] Krauss E, Kullock R, Wu X, et al. Controlled Growth of High-Aspect-Ratio Single-Crystalline Gold Platelets. Crystal Growth & Design 2018;18:1297–302.

[2] Celebrano M, Wu X, Baselli M, et al. Mode matching in multiresonant plasmonic nanoantennas for enhanced second harmonic generation. Nature Nanotechnology 2015;10:412–7.

[3] Vieu C, Carcenac F, Pépin A, et al. Electron beam lithography: resolution limits and applications. Applied Surface Science 2000;164:111–7.

[4] Joshi-Imre A, Bauerdick S. Direct-Write Ion Beam Lithography. Journal of Nanotechnology 2014;2014:1–26.

[5] Horák M, Bukvišová K, Švarc V, et al. Comparative study of plasmonic antennas fabricated by electron beam and focused ion beam lithography. Scientific Reports 2018;8:9640.

[6] Klein MW, Wegener M, Feth N, et al. Experiments on second- and third-harmonic generation from magnetic metamaterials. Optics Express 2007;15:5238–47.

[7] Feth N, Linden S, Klein MW, et al. Second-harmonic generation from complementary split-ring resonators. Optics Letters 2008;33:1975–7.

[8] Niesler FBP, Feth N, Linden S, et al. Second-harmonic generation from split-ring resonators on a GaAs substrate. Optics Letters 2009;34:1997–9.

[9] Jeyaram Y, Verellen N, Zheng X, et al. Rendering dark modes bright by using asymmetric split ring resonators. Optics Express 2013;21:15464.

[10] O’Brien K, Suchowski H, Rho J, et al. Predicting nonlinear properties of metamaterials from the linear response. Nature Materials 2015;14:379–83.
